# Effects of resveratrol pretreatment on endoplasmic reticulum stress and cognitive function after surgery in aged mice

**DOI:** 10.1186/s12871-018-0606-5

**Published:** 2018-10-10

**Authors:** Bei Wang, Shengjin Ge, Wanxia Xiong, Zhanggang Xue

**Affiliations:** Department of Anesthesia, Zhongshan Hospital, Fudan University, Shanghai, 200032 China

**Keywords:** Resveratrol, Endoplasmic reticulum stress, Cognitive function, Surgery, Aged mice

## Abstract

**Background:**

Postoperative cognitive dysfunction (POCD) seriously reduces quality of life and is associated with increased morbidity and mortality. The causes and neuropathogenesis of POCD remain largely unknown. Resveratrol, a sirtuin 1 (Sirt1) activator, is a polyphenol compound found in red wine that has protective functions in neuropathology paradigms. Endoplasmic reticulum stress (ERS) is a primary cellular response that activates the unfolded protein response (UPR). ERS and UPR mediate molecular and biochemical mechanisms related to neurodegeneration; however, the roles of ERS and Sirt1 in POCD remain unclear. The properties of resveratrol might be useful in the setting of POCD.

**Methods:**

In the present study, we investigated learning and memory function and ERS pathways in aged mice after surgery under local anesthesia, and we evaluated the effects of resveratrol pretreatment.

**Results:**

We found that resveratrol attenuated postoperative learning and memory impairment in aged mice postoperatively but did not alter locomotor activity. Resveratrol significantly decreased postoperative expression of ERS pathway UPR-related proteins and inflammatory mediators including nuclear factor-κB (NF-κB) in the hippocampus. This was accompanied by higher Sirt1 protein expression levels. Pretreatment with resveratrol did not affect the number of hippocampal neurons in aged mice after surgery.

**Conclusion:**

Overall, resveratrol pretreatment attenuated short-term learning and memory impairment and the ERS pathway UPR in aged mice after surgery under local anesthesia.

## Background

Aged patients are prone to postoperative decreases in learning and memory function, resulting in decreased quality of life and increased social burden [1–3]. Improvements of anesthesia and surgical techniques have led to larger numbers of older patients receiving anesthesia and surgery. These patients are more likely to exhibit postoperative learning and memory impairment.

Annually, there are approximately 2.5 million surgical operations worldwide [4]. Improving postoperative cognitive function and preventing postoperative cognitive dysfunction (POCD) are therefor of great significance. The factors of POCD are extremely complex [5, 6], but recent studies have focused on the impacts of anesthesia factors and general anesthesia drugs, especially inhalation agents such as isoflurane and sevoflurane, on the central nervous system [7–10]. Patients undergoing general or even regional or local anesthesia for surgical treatment can experience POCD [11, 12]. One group found that rats under intravenous anesthesia during right carotid artery exploration still exhibited POCD compared with inhalation anesthesia [13]. It is important to study the effect of surgery on the central nervous system and neuronal inflammation to clarify the mechanism(s) of POCD and identify appropriate interventions.

Endoplasmic reticulum stress (ERS) is the initial response of cells under stress [14, 15]. ERS has been linked with cognitive function in inhaled anesthetics-induced cognitive dysfunction and neurodegenerative conditions such as Alzheimer’s disease [16, 17]. It is suggested that ERS played an important role in surgery-induced cognitive impairment in animal models and ERS could be attenuated by some intervening [18, 19].. The unfolded protein response (UPR) in the ERS pathway involves three membrane proteins that are important endoplasmic reticulum receptors: PERK (the PKR-like ER protein kinase), IRE1 (the inositol-equiring enzyme 1), and ATF6 (the activating transcription factor 6). The released BiP/GRP78 activates important downstream pathways including PERK/EIF2α, IRE1 /XBP1, and ATF6 [20]. IRE1 in the UPR is a highly conservative transmembrane protein that localizes to the endoplasmic reticulum and can activate including nuclear factor-κB (NF-κB) [21]. C/EBP homologous protein (CHOP) is thought to be a pro-apoptotic transcription factor and plays a critical role in ERS-induced apoptosis [22].

Resveratrol is found naturally in grapes, mulberry, and other plants. It is a natural polyphenol and can activate sirtuin 1 (Sirt1). It has antioxidant effects and affects downstream signaling pathways [23]. Numerous in vivo and in vitro studies have demonstrated that resveratrol can protect the myocardium and exert anti-tumor, anti-oxidative, anti-inflammatory, and neuroprotective effects by activating Sirt1 [24–27].

However, the effect of resveratrol on the ERS in POCD is not clear. The goal of this study was to investigate whether resveratrol pretreatment affects learning and memory function in aged mice after surgery. We examined the effects on the ERS-related inflammatory marker NF-κB and ERS-related UPR-specific proteins such as PERK, GRP78, CHOP, XBP1u, and IRE1α.

## Methods

### Animals

The experiments utilized 18-month-old male C57BL/6 mice that were housed at the Animal Experimental Center of Zhongshan Hospital, Fudan University. The mice were housed in cages at a temperature of 22 °C for 12 h. All the experimental protocols were approved by the animal ethics committee of Zhongshan Hospital Affiliated to Fudan University, China. Reference number for the ethics approval is 2016–0130. Mice were randomly divided into three groups: resveratrol (*n* = 10), surgery (*n* = 10), and control (*n* = 10). Mice in the resveratrol group were pretreated with resveratrol for 7 days at a dose of 100 mg/kg via intraperitoneal injection. The mice in the surgery group were given 7 days of the same amount of vehicle intraperitoneally. Mice in the resveratrol and surgery groups underwent exploratory laparotomy under local anesthesia on the 7th day after intraperitoneal injection. Mice in the control group received 7 days of intraperitoneal vehicle without surgery.

### Surgical model

The mice model in our study was modified from the model in series studies explored by Xie C [28, 29]. Mice were gently fixed with a paper tape on a plastic separator, and covered with a 37 °C constant temperature electric blanket. All surgical instruments were sterilized by autoclaving. Bupivacaine (0.5%, 0.1 ml) was applied to the skin and subcutaneous tissue in the abdominal region of each mouse. A 1.5 cm incision was made parallel to the median line on the right side of the midline, followed by gentle abdominal exploration. Then the muscle fascia and skin were sutured with 3–0 sterile silk. Surgery lasted 5 min, without sedative drugs or antibiotics. Each mouse was operated on with sterile instruments and newly opened sterile sutures. All mice breathed spontaneously throughout surgery, and we monitored their respiration rate, limb color, and temperature. Postoperatively, the mice were returned to the original environment to continue feeding with regular observation of wounds and vital signs. Saline was used once per day for 2 or 7 days post-surgery to avoid the infections.

### Open field test

Locomotor activity was tested in the open field at 1 day postoperatively. The mice were moved from the home cage to the corner of the open field (120*120 cm), which was divided into a grid of 3*3 squares. Animal movement in the area was recorded for 10 min. The total distance traveled and mean speed were recorded and quantified with Any-Maze software.

### Fear conditioning system (FCS)

The animals received FCS pairing training 24 h postoperatively. At 48 h and 7 days after surgery, the animals were subjected to context and tone testing.

Behavioral testing using FCS was divided into two stages of training and testing. The training phase was performed by placing the mouse in a test chamber (30 cm * 30 cm * 45 cm) for 180 s, giving a 2-Hz 60-s sound stimulation, then a foot shock immediately after the end of the stimulus (0.8 mA for 0.5 s). This training process has two consecutive periods, with a 2-min middle interval for a rest.

The first context test was performed 30 min after the end of the training, and each mouse could remain in the same chamber for 390 s. Learning and memory evaluation for the context test was a percentage of the lag time, and freeze was defined as the percentage of time spent not moving in the second 180-s period. The first tone test was performed 90 min after the end of the training. Each mouse could stay in the same chamber for 390 s. The same tone was played in the second 180 s, but without a foot shock. The learning and memory evaluation in the tone test was the freezing time percentage. The same procedures for the context and tone tests were performed 48 h and 7 days after surgical intervention.

At the end of the experiment, the mice were returned to their home cage for feeding. After testing each mouse, the site was wiped with 75% alcohol to eliminate smell interference.

### Nissl staining

After the FCS at 7 days postoperatively, the mice (*n* = 4 in each group) were deeply anesthetized and perfused with normal saline, followed by 4% paraformaldehyde. Brains were fixed, dehydrated in a sucrose gradient, and series sectioned. The section thickness was 20 μm. Images were taken at 400 times magnification, for 75 fields per group. The numbers of hippocampal neurons were calculated.

### Western blot

After the FCS at 7 days postoperatively, the mice (*n* = 6 in each group) were sacrificed under deep anesthesia. The hippocampal tissues were removed out and then immediately placed in liquid nitrogen and subsequently stored at − 80°Cuntil tissue homogenization. The expression of Sirt1 and ERS unfolding protein-related proteins were detected by western blot. Samples were centrifuged, and protein concentrations were determined with the bicinchoninic acid method. Equal amounts were then subjected to sodium dodecyl sulfate-polyacrylamide gel electrophoresis, transferred to membranes, and incubated with appropriate antibodies. Western blotting assay was performed six times. β-actin was used as the internal reference (Santa Cruz); other antibodies were Sirt1 (Santa Cruz), IRE1α (Cell Signaling Technology), PERK (Cell Signaling Technology), XBP1u (Santa Cruz), CHOP (Cell Signaling Technology), GRP78 (Santa Cruz), and NF-κB (Cell Signaling Technology). Each experiment was repeated at least four times. Integrated density values of specific proteins were quantified using ImageJ software. Relative expression levels of proteins were normalized to β-actin.

### Real-time PCR

Total RNA was isolated from homogenized hippocampal tissues using the Trizol extraction method. Real-time quantitative PCR was used to detect mRNA expression. β-actin was used as an internal control. The primers were Sirt1 sense strand CAGCATCTTGCCTGATTTGTAA, antisense strand TGGGGTATAGAACTTGGAATTAGTG; IRE1α sense strand AGAATCAGACGAGCACCCAAATG, antisense strand GAGAATGTTGTGGGGCTTCAGGT. The relative expression of mRNA was quantified using the 2–ΔΔCt method. The results were normalized to β-actin as reference gene.

### Statistical analysis

**Fig. 1:**
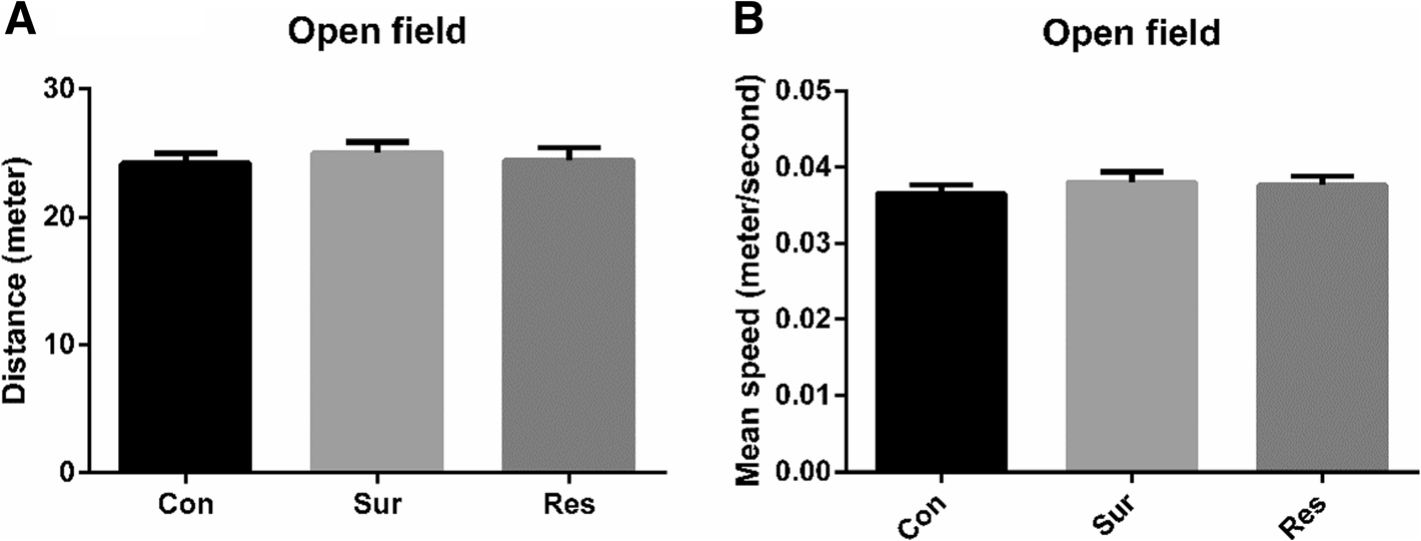
The distance (**a**) and mean speed (**b**) in the open field test 1 day postoperatively; Con, control; Res, resveratrol; Sur, surgery. The number of mice in each group was 10

**Fig. 2:**
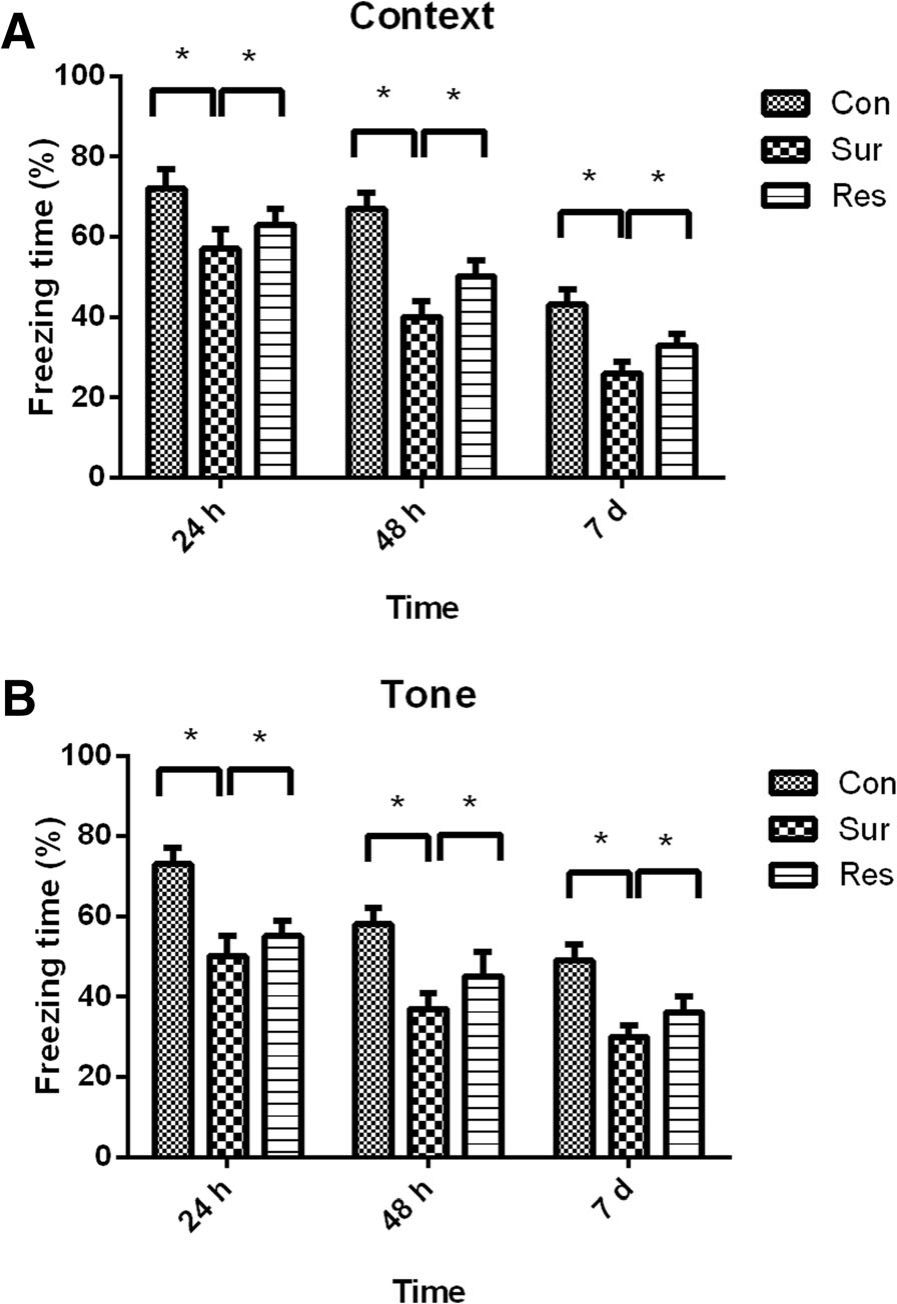
The freezing time percentages for the context (**a**) and tone (**b**) tests at 24 h, 48 h, and 7 days postoperatively; Con, control; Res, resveratrol; Sur, surgery. *P < 0.05. The number of mice in each group was 10

**Fig. 3:**
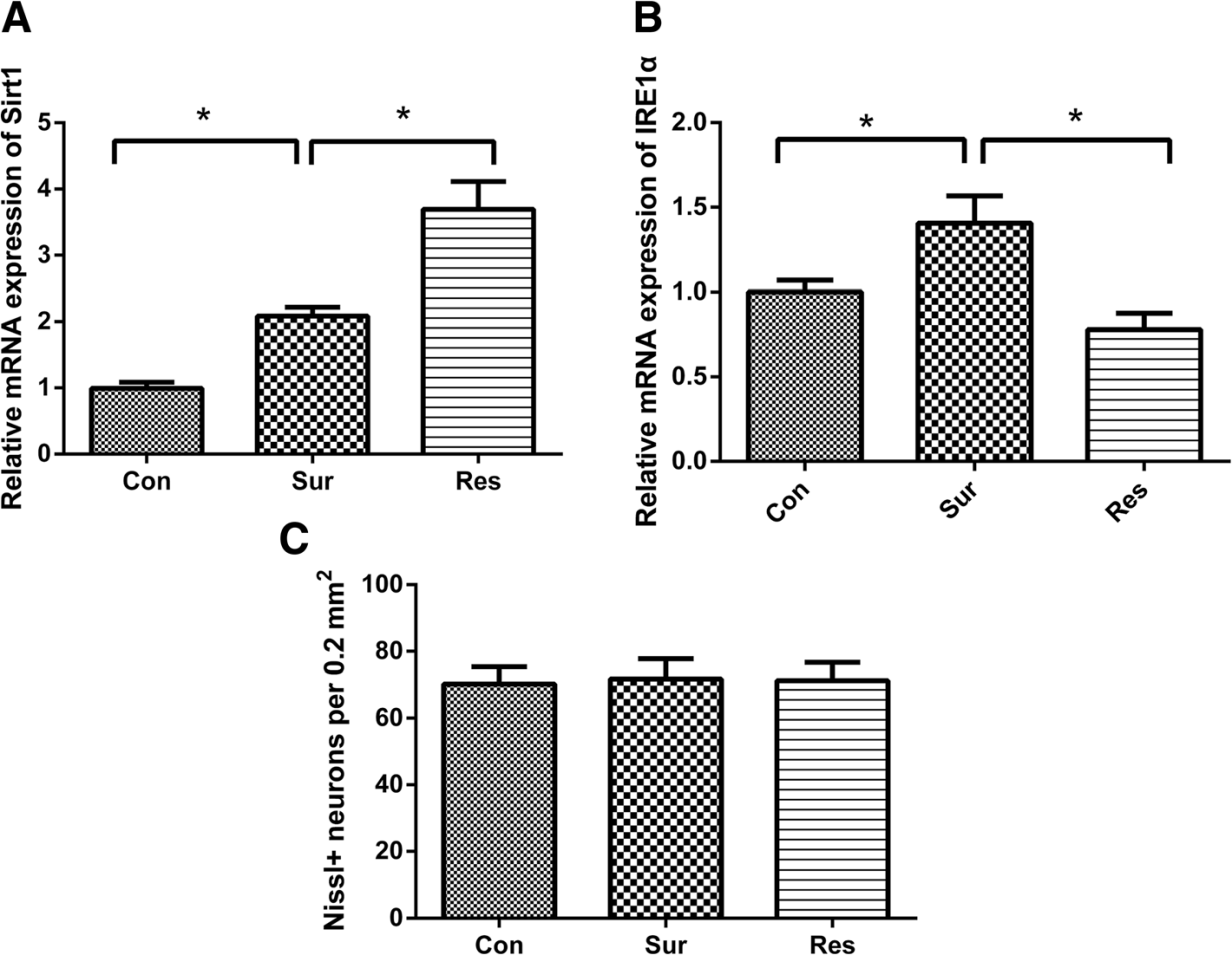
Sirt1-mRNA (**a**) and IRE1α-mRNA (**b**) expression in the hippocampus and CA1 neuron density (**c**) at 7 days postoperatively; All values were expressed as fold changes over the mean values of control and were presented as mean ± SD (*n* = 4). *P < 0.05. Con, control; Res, resveratrol; Sur, surgery

**Fig. 4:**
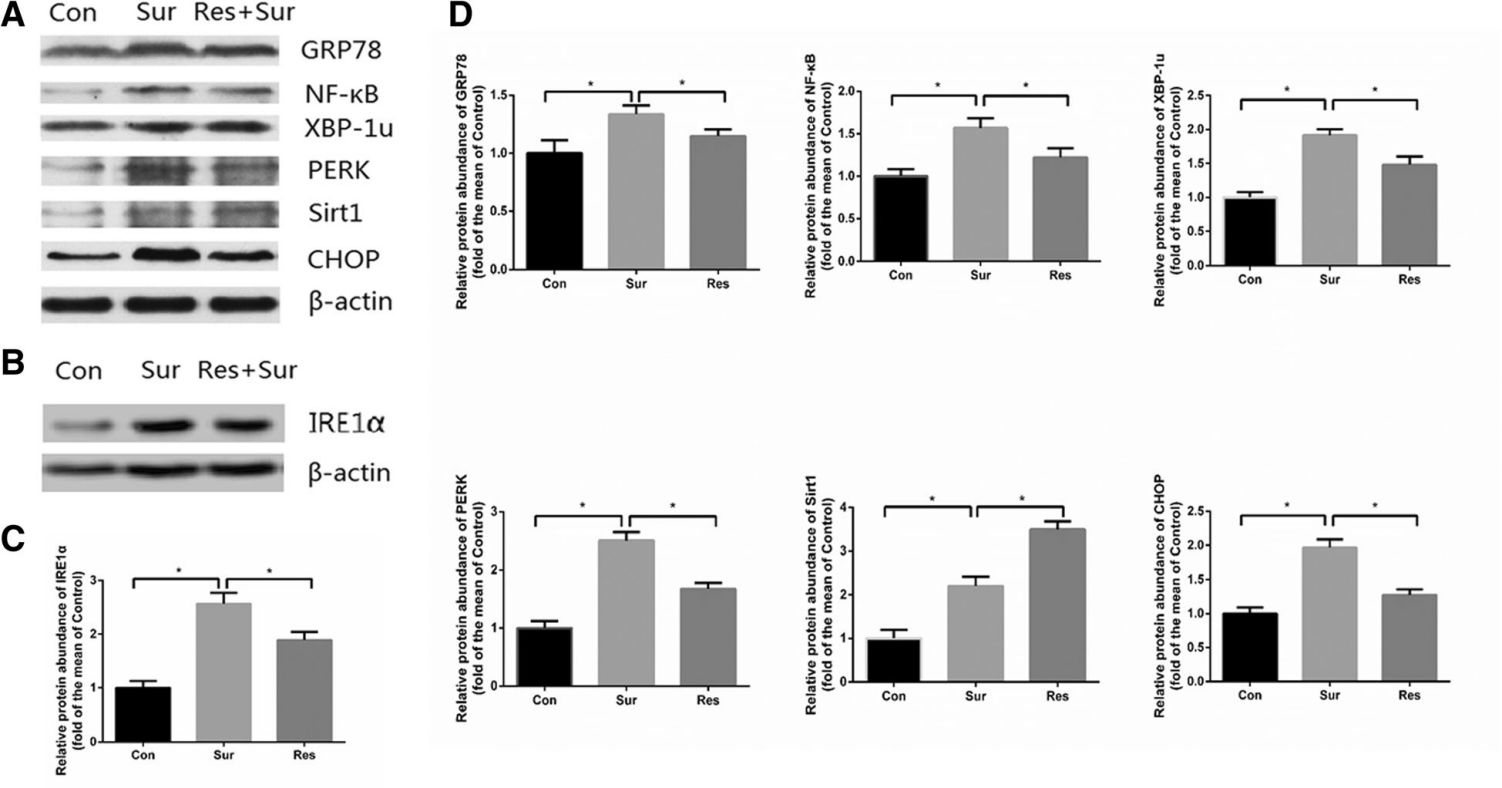
Sirt1 and unfolded protein response-related proteins IRE1α, PERK, XBP1, CHOP, GRP78, and NF-κB in the hippocampus (**a**, **b**) Western blot results and (**c**, **d**) relative protein expression levels at 7 days postoperatively. All values were expressed as fold changes over the mean values of control and were presented as mean ± SD (*n* = 4). *P < 0.05. Con, control; Res, resveratrol; Sirt1, sirtuin 1; Sur, surgery

SPSS19 software was used to analyze the data for normality and variance homogeneity. Data are expressed as mean ± standard deviation. The results were compared using two-way analysis of variance in Fig. 2. The results were compared using one-way analysis of variance in Figs. 1, 3 and 4. Two-tailed tests were employed in all comparisons. Differences were considered significant at *p* < 0.05. Graphs were created using GraphPad Prism 6.

## Results

### Neither surgery nor resveratrol pretreatment affected locomotor activity

Surgery did not significantly affect the distance or mean speed in the open field test. Similarly, resveratrol did not significantly affect the distance or mean speed compared with the surgery group (Fig. 1a and b).

### Pretreatment with resveratrol attenuated the POCD in aged mice

Compared with the control group, the freezing times for both the context and tone tests at 24 h, 48 h, and 7 days postoperatively were significantly decreased in the surgery group. The freezing times of the resveratrol group were significantly higher than that of surgery group (Fig. 2a and b). These results indicated that pretreatment with resveratrol attenuated learning and memory impairment in aged mice.

### Pretreatment with resveratrol increased hippocampal Sirt1 and Sirt1-mRNA expression

The protein and mRNA levels of Sirt1 in the surgery and resveratrol groups were significantly different compared with those in the control group (Figs. 3a, 4a and d). Western blot analysis revealed that Sirt1 levels were significantly higher in the resveratrol pretreatment group compared to the surgery group (Fig. 4a and d). Real-time PCR showed greater Sirt1-mRNA expression in the resveratrol pretreatment group compared to the surgery group (Fig. 3a). This indicated that resveratrol preferentially stimulated Sirt1 expression in the hippocampus.

### Resveratrol attenuated learning and memory impairment in aged mice

After the final behavioral test at 7 days postoperatively, hippocampal sections were subjected to Nissl staining. There was no significant difference in hippocampal neuron density between the resveratrol and surgery groups (Fig. 3c). This indicated that resveratrol had no effect on the number of neurons in the hippocampus.

### Pretreatment with resveratrol attenuated ERS-induced UPR in the hippocampus of aged mice

Western blot analysis showed that the expression levels of UPR-related proteins in the surgery group were significantly increased than the control group. The expression levels of UPR-related proteins in the resveratrol group were significantly different from the other two groups. Compared with the surgery group, the expressions of CHOP, IRE1α, XBP1u, PERK, and NF-κB were significantly decreased in the resveratrol group (Fig. 4). Real-time PCR results showed significantly lower hippocampal IRE1α mRNA levels in the resveratrol pretreatment group compared to the surgery group (Fig. 3b). These indicated that pretreatment with resveratrol could attenuate surgery induced UPR and reduce the expression of inflammatory mediators.

## Discussion

Mice receiving exploratory laparotomy under local anesthesia exhibited learning and memory impairment. This demonstrates that surgery induced cognitive dysfunction in aged mice without general anesthesia or inhalation anesthetics [28]. The expression levels of Sirt1 and UPR-related proteins in the hippocampus were significantly different between the surgery and control groups. Sirt1 expression was increased in the surgery group and could play an important role in the regulation of cellular functions under stress, but it was not associated with a neuroprotective effect yet. The result of present study was consistent with some studies. [30].

The results confirmed that resveratrol pretreatment of aged mice prior to local anesthesia could activate hippocampal expression of Sirt1. The effects of resveratrol have been studied and reported in several diseases models, but the numerous pathways it influences remain to be fully elucidated. Resveratrol can directly activate Sirt1 expression at transcriptional and translational levels, and it exerts its deacetylation pathway downstream [31–33]. However, recent studies have shown that many mediators are involved in resveratrol-induced Sirt1 expression, suggesting that resveratrol both directly and indirectly activates Sirt1 [34, 35].

Resveratrol exert anti-tumor, anti-oxidative, anti-inflammatory, and neuroprotective effects by activating Sirt1. Resveratrol has been suggested to enhance/increase ER stress in cancer cells [36–38]. The specific mechanism of resveratrol anti-tumor and neuroprotective effects remain unknown. Several studies have proved that resveratrol could suppress ER stress in heart and brain [39, 40]. We found that resveratrol pretreatment attenuated postoperative learning and memory impairment in aged mice. The mechanism of resveratrol in cognitive function is very complex, probably independent of the role of Sirt1 [41–43]. The present findings show the possibility that ERS suppression could be one of the mechanisms.

The results indicated that resveratrol pretreatment could attenuate the ERS pathway UPR in the hippocampus of aged mice under local anesthesia. Resveratrol ameliorates ERS by many mechanisms, such as downregulating CHOP and GRP78 gene expression and attenuating caspase-3 activity and ERS could be mediated by some mechanisms as well [44]. Resveratrol was shown to prevent doxorubicin-induced cardiotoxicity in H9c2 cells by inhibiting ERS and Sirt1 pathway activation [39]. ERS could be involved in cognitive function. Amelioration of ERS may therefore serve as a novel strategy to alleviate cognitive function triggered by microcystin-leucine-arginine [45]. Honokiol abrogates chronic restraint stress-induced cognitive impairment and depressive-like behavior by blocking ERS in the mouse hippocampus [46].

Although our results show that resveratrol can alleviate ERS in a POCD model, the specific pathway remains unclear. The target for ER-specificity is unknown, and the mechanisms of ER-related pathways need to be elucidated [47, 48]. Amelioration of ERS could be explored in further studies. Resveratrol pharmacokinetics still need to be investigated to identify the appropriate dose. The anti-inflammatory and anti-apoptotic effects of resveratrol on ERS and other mechanisms of action are interrelated and need to be clarified. Specifically, it will be important to determine whether resveratrol can affect cognitive function in animals treated with antagonists of ERS. The relationship among resveratrol, Sirt1, ERS and cognitive function remain further study. We would focus on the association between ER stress and Sirt1 in vitro and in vivo in our following study.

## Conclusion

In summary, resveratrol pretreatment could activate Sirt1 expression and reduce postoperative ERS in aged mice. Resveratrol also attenuates POCD. These results reveal an important role of ERS in surgery under local anesthesia and provide a potential study target for POCD.

## Abbreviations

ATF6: The activating transcription factor 6
CHOP: C/EBP homologous protein
ERS: Endoplasmic reticulum stress
FCS: Fear conditioning system
IRE1: The inositol-equiring enzyme 1
NF-κB: Nuclear factor-κB
PERK: Rhe PKR-like ER protein kinase
POCD: Postoperative cognitive dysfunction
Sirt1: Sirtuin 1
UPR: Unfolded protein response
